# Exergy Analysis of the Heart with a Stenosis in the Arterial Valve

**DOI:** 10.3390/e21060563

**Published:** 2019-06-04

**Authors:** Julio Brandão Roll, Matheus Leone Borges, Carlos Eduardo Keutenedjian Mady, Silvio de Oliveira Junior

**Affiliations:** 1 Polytechnic School of the University of São Paulo, Av. Luciano Gualberto 380, São Paulo 05508-010, Brazil; 2 School of Mechanical Engineering, University of Campinas, Mendeleyev St. 200, Campinas 13083-970, Brazil

**Keywords:** exergy analysis, cardiovascular system, heart, human body, aortic stenosis

## Abstract

In the past decade, several articles have proposed the use of an exergy perspective to analyze physiological systems of the human body under different physical conditions. Such a perspective focuses on the exergy transformations and the efficiency of the biological processes. This may aid the medical field in assessments of a patient’s physical health by means of an index (exergy efficiency) based on the quality of the energy conversion in a given process within the human heart. As a follow-up, a model was developed to describe the evolution of the transvalvular pressure gradient in the aortic valve as a function of stenosis severity. This model was created using physiological data from 40 patients available in the literature, as well as 32 operating points from different bileaflet aortic valve prosthesis. A linear regression results in values around 14.0 kPa for the pressure gradient in the most severe case, evolving from 1.0 kPa for a healthy scenario. The thermodynamic model assesses the irreversibilities associated with energy conversion processes related to metabolism: exergy destroyed at the valves, exergy increased in the flow, and the power of the heart. Results indicate that destroyed exergy reaches values of 10 W (almost 10% of total basal metabolic rate of the whole body). Exergy efficiency is 15% for a healthy heart, decreasing as a function of the severity of the stenosis to values lower than 5%.

## 1. Introduction

Any energy conversion process can be analyzed through the application of the First and Second Laws of Thermodynamics. While the former states that energy is conserved in every process, the latter quantifies the amount of irreversibility of any process. One approach to the Second Law of Thermodynamics is the use of exergy. The term exergy was proposed for the first time by Rant [1] and later by Szargut et al. [2], who defined exergy as a physical quantity capable of characterizing the quality of any kind of energy conversion process. In this way, exergy analysis presents itself as a useful tool to determine the losses and opportunities for improvement in a system or process using the same scale, i.e., the capacity of any system to perform work.

Towards a proper understanding of how the human body functions, one of the most fascinating and challenging applications of the Second Law of Thermodynamics is the understanding of biological systems at all levels of organization, from cells to living organisms [3]. The exergy analysis of the human body and its organs can deepen the understanding of energetic processes that may be unclear to the medical field, enhancing our knowledge of how aging and pathologies may affect the behavior of the body.

The study of specific organs and systems of the human body is a focus of several literature publications. Applications have ranged from the microlevel with cancerous cells [4,5], neurons [6], and muscles [7], to the macrolevel with the human respiratory system [8], the heart [9,10], the whole body [11,12,13,14], treatment for patients with severe burns [15], and even an entire society of individuals [16]. Regarding the evaluation of an organ from the Second Law perspective, the authors of [9] proposed a model that divides the heart into two main pumps, referred to as the left and right hearts. Hence, it is possible to obtain the destroyed exergy of a healthy heart and an unhealthy one. Moreover, the authors of [10] carried out studies on the exergy behavior of a normal sized heart and the effect of aging with a reduction in nutrient consumption of the heart. Regarding the analysis of pathology treatments, the authors of [15] concluded that the lowest exergy consumption leads to optimal healing conditions of patients affected by severe burns.

One of the pioneering applications of the Laws of Thermodynamics to the heart was carried out by Blick and Stein [17]. The authors assessed the contributions of different terms to energy conservation. Henriques et al. [9] analyzed the human heart and its destroyed exergy under rest, walking, and running conditions for both normotensive and hypertensive subjects. As exercise intensity increased in the presence of hypertension, the authors observed an increase in total destroyed exergy in the heart which was mainly due to a higher pumping pressure and increased metabolic rate. Moreover, as expected, the destroyed exergy in the left part of the heart is substantially higher than that of the right one since the former is responsible for providing blood flow for the systemic circulation (whole body).

Recently, the effects of reductions in the blood flow rate due to obstructions were studied by [18,19]. These authors expanded the field of exergy analysis by considering pathological cases such as an analysis of the destroyed exergy in an artery due to an obstruction in the aortic artery [18] and a stenosis in the arterial valve of the heart [19]. Both articles considered the constriction in the artery as a flow singularity, similar to industrial pipes, causing head loss and, thus, irreversibilities.

## 2. Exergy Analysis Methodology Applied to the Human Heart

**Figure 1:**
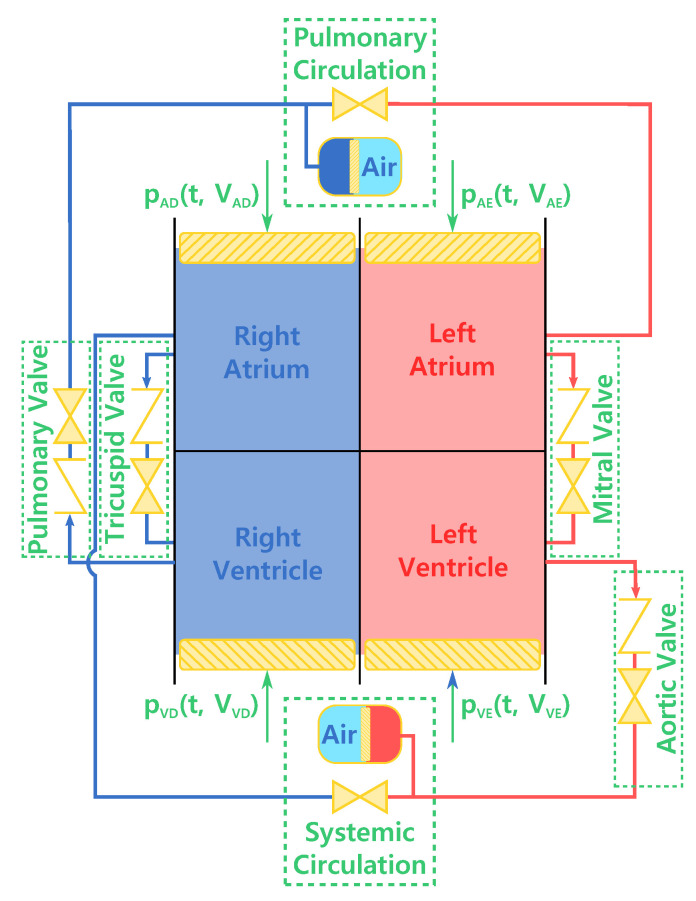
Thermodynamic model of the heart, taking into account the head loss in valves and in the pulmonary and systemic circulations. Based on [21].

A thermodynamic model was proposed in order to assess the phenomenological and process behavior of the human heart. This model is summarized in Figure 1, where the heart is represented as four chambers. The left side is composed of an atrium and a ventricle with the mitral valve separating both cavities (with the respective head loss). From the left ventricle, the blood passes through the arterial valve (with head loss), and is sent to the systemic circulation. The same process happens on the right side, where blood flows through the tricuspid valve, enters the right atrium, and passes through the pulmonary valve in order to go to the pulmonary circulation, where it exchanges carbon dioxide and oxygen with the inspired air [8]. These two circulations have a vascular resistance of $R_{pul}$ = 7.44 MPa · s/m^3^ (0.93 mmHg · min/L) and $R_{sys}$ = 140.32 MPa · s/m^3^ (17.54 mmHg · min/L) [19,20], respectively. This physical quantity can be measured using the pressures in all the four cavities of the heart and the volumetric blood flow. However, in this study the focus is on the human heart, which is where we place our control volume.

### 2.1. Phenomenological Model

**Figure 2:**
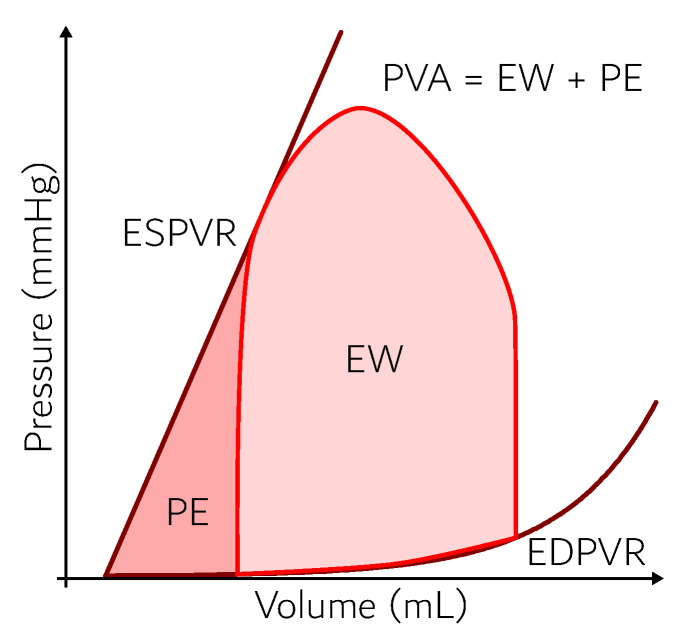
Schematic representation of the cardiac cycle (ventricular pressure–volume diagram). Adapted from [22].

As discussed in [22], the heart may be considered as a pump that converts mechanical, chemical, and electrical energy into heat and flow work. Figure 2 indicates the cardiac cycle, which consists of an isovolumetric increase in the pressure, a systolic ejection, an isovolumetric relaxation, and a filling period. The area confined by the cardiac cycle is known as external work of the heart ($EW={\dot{W}}_{Heart}$) and may be calculated by means of Equation (1) [23]. According to Schaldach [24], the muscle cells of the heart contract by electrical activity originating in specialized cells of the myocardium that serve as the heart pacemakers. The same author states that the voltage gradient across the cell membrane is about −80 mV at resting state. This value can be modified when ions such as sodium, potassium, and calcium flow through the membranes of the muscles cells. By this process, the potential difference may reach values as high as +30 mV with an electrical current of 0 to 5 mA [24]. In order for the heart to perform any movement, there must be a trigger to stimulate its contraction [17].

(1) $${\dot{W}}_{Heart}=\int_{V_{min}}^{V_{max}}p\left(V\right)dV$$

The remaining shaded area in Figure 2 is the potential energy (PE). The dark lines are obtained experimentally from the filling of an isolated heart and are known as the end diastolic and systolic volumes. The full area is the pressure–volume area (PVA), and it is used as a basis to calculate the oxygen consumption of the organ [23]. The expression for the PVA is shown in Equation (2).

(2) $$PVA={\dot{W}}_{Heart}+\dot{PE}$$

According to [22,25], it is possible to correlate the oxygen consumption of the myocardium with the PVA in Figure 2. Experimental results obtained in canine hearts [25] identified a linear correlation between the PVA and oxygen consumption. The authors proposed, then, that one can calculate the ${\dot{m}}_{V_{O_{2}}}$ using the pressure-volume area. This linearity was studied by other authors such as [26,27,28]. The benefit of this type of analysis is the possibility to extrapolate this result to hearts under different conditions.

Therefore, the rate in which the myocardium consumes oxygen, or ${\dot{m}}_{V_{O_{2}}}$, can be calculated from a linear correlation using the PVA. The authors [27] proposed the linear expression for human beings shown in Equation (3) with a correlation coefficient of 0.991.

(3) $${\dot{V}}_{O_{2}}=\left(1.82\cdot 10^{-5}\right)PV\;A+0.0284$$

From the results of Equation (3) and the results obtained in [23], the production of CO_2_ can be obtained from the molar respiratory relations, bearing in mind that 30% of the nutritional consumption in the heart is from carbohydrates (represented by glucose with with RQ=1), and 70% is from lipids (represented by palmitic acid with RQ=0.696). It was assumed that the ratio of carbon dioxide production and oxygen consumption on a molar basis (RQ) is 0.787. The correlations obtained in [29] demonstrate how to quantify the metabolism on an energy and exergy basis according to Equations (4) and (12).

(4) $${\dot{M}}_{Heart}=11371{\dot{m}}_{O_{2}}+2366{\dot{m}}_{CO_{2}},$$

where ${\dot{m}}_{O_{2}}$ and ${\dot{m}}_{CO_{2}}$ are the oxygen consumption and carbon dioxide production of the human heart, respectively.

Part of the energy of the metabolism is used to perform work, while the other share is released as heat as shown in Equation (5). Therefore, the heat released by the metabolism is only equal to the metabolic activity if there is no workload.

(5) $${\dot{Q}}_{M_{Heart}}={\dot{M}}_{Heart}-{\dot{W}}_{Heart},$$

where ${\dot{M}}_{Heart}$ is the energy metabolism, ${\dot{Q}}_{M_{Heart}}$ is the heat associated with the metabolism, and ${\dot{W}}_{Heart}$ is power.

**Figure 3:**
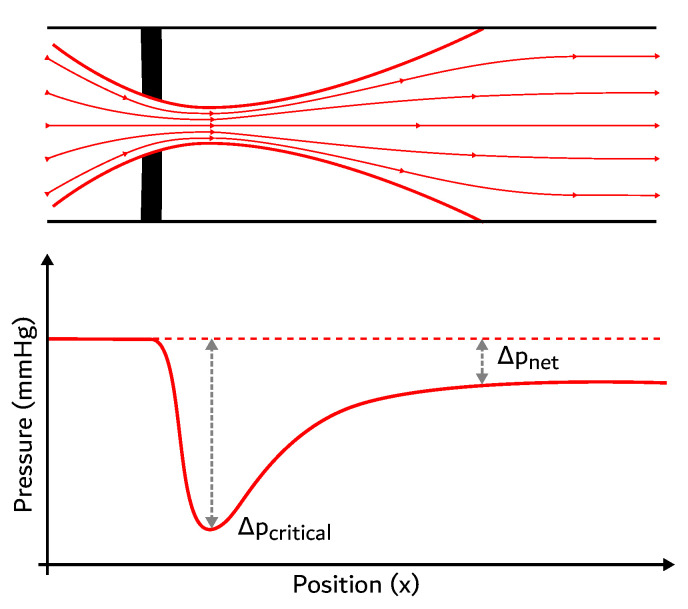
Representation of a flow in a valve. Adapted from [19].

According to [19], after taking into account the head loss due to friction in the blood vessels (which is not the focus of the present work), it is also necessary to account for the minor loss that happens when flow passes through the heart. This is predominantly caused by the four valves of the heart (other phenomena, such as recirculation, will be ignored here). In order to quantify such loss, Gorlin and Gorlin [30] proposed a framework whose use is widespread in the hemodynamics literature for studying the heart valves. The authors proposed an orifice plate view of the valves, represented in Figure 3, which also shows the evolution of the transvalvular pressure gradient (TPG) in the fluid. The gradient reaches its peak at the vena contracta, a point where the cross-sectional area of the flow is minimal (called the EOA: effective orifice area). Figure 3 represents the flow through a valve based on the equations demonstrated by [30].

Equation (6), also known as the “Gorlin equation” [30], is derived from Figure 3. In this equation, AVA represents the area of the arterial valve, *C*, $C_{v}$, and $C_{c}$ are constants [30], $\rho_{bl}$ is the specific mass of the blood, ${\dot{V}}_{syst}$ represents the volumetric flow rate of blood, HR stands for the heart rate, $t_{E}$ is the time at which the ventricle or atrium ejects blood, and ΔP represents the pressure drop across the valve (TPG—mmHG). For simplicity, a constant *K* (Equation (7)) can be used to calculate Equation (6). This constant is equal to 44.3 for the aortic valve and 37.7 for the mitral valve, both in $cm/s\sqrt{mmHg}$ [30,31].

(6) $$AVA=\frac{1}{CC_{v}C_{C}}\sqrt{\frac{\rho_{bl}}{2}}\frac{{\dot{V}}_{syst}}{HRt_{E}}\frac{1}{\sqrt{\Delta P}},$$

(7) $$K\propto C_{v}C_{c}\sqrt{\frac{1}{\rho_{bl}}}.$$

In this framework, the input and output of both sides of the heart (hereafter, the “right” and “left” hearts) are considered to be one single flow with the cross-sectional area of the valve as the sum of the areas on both sides. For the right heart, the areas of the tricuspid valve and pulmonary valve were taken from [32,33], respectively. For the left heart, we adopted 5 cm^2^ for the mitral valve area (MVA) and a range of 0.1 to 4.0 cm^2^ for the aortic valve area [19].

**Figure 4:**
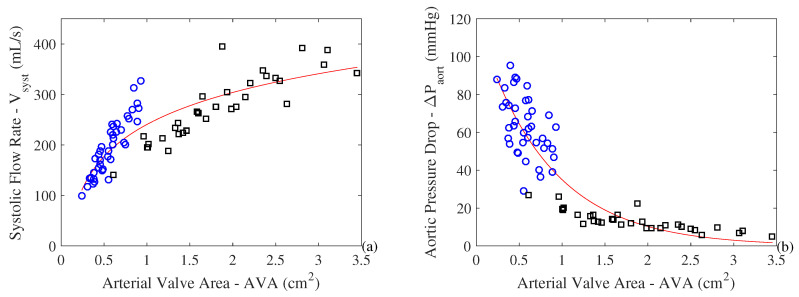
Logarithmic linear regression, Equation (8), between the systolic volume flow (**a**) and pressure drop (**b**), for the the aortic valve area measured in 40 patients with stenosis (blue circles) [34] and 32 points of operation of prostheses representing healthy patients or a less severe stenosis (black squares) [36]. R^2^ of 0.7497. Retrieved from [19].

Using the results of [34], we ran a linear regression using the data for 40 patients with severe stenosis. However, in order to properly analyze the effect of the evolution of the pathology on the exergy analysis, one requires data on healthy subjects or values of pressure drop and cardiac output close to operational conditions of healthy hearts. Probably due to a lack of interest in studying the correlation between healthy cardiac output and valve area, such data are not readily available in the literature. Hence, an additional 32 points were added from patients with prosthetic valves under different operating conditions. Our goal using these points was to obtain a pressure drop behavior close to that of healthy original valves. These prosthetic valves are called *bileaflet*, and their operational data were obtained from the MEDLINE data in [35]. This type of prosthesis is well known in the medical field, and its use has been certified for different operating points. Therefore, data from healthy and sick patients were used as indicated in Figure 4a for the systolic flow rate. The linear regression given by Equation (8) represents the volumetric flow (or cardiac output) as a function of the area of the aortic valve. Using Equations (6) and (7), we assessed the aortic pressure drop (ΔP), or TPG.

Regarding the range of values for the AVA, the authors of [34] found that the range was from 0.24 to 0.93 cm^2^ for patients with severe stenosis in the aortic valve, which matches the reference value in [20] for the development of symptoms due to the stenosis (i.e., AVA < 1.0 cm^2^). For healthy patients and those with mild pathological conditions, AVA ranged from 0.61 to 3.45 cm^2^ [35]. Moreover, these data are in accordance with medical literature such as [37]. The authors argue that the range from 3 to 4 cm^2^ represents a healthy aortic valve area. It is important to highlight that reductions in the aortic valve area do not represent a modification in the pressure drop until a certain value of severity. Therefore, the range of the prosthesis data (healthy or with small stenosis) could be used according to Figure 4 to represent the behavior of the valve from a healthy scenario to a stenotic one. Some authors [38] recommend considering magnitudes of AVA larger than 1.2 cm^2^ as normal, between 1.2 and 0.8 cm^2^ as a possible case of stenosis, and lower than 0.8 cm^2^ as an indication of significant stenosis. More information regarding these differences can be found in [36].

It is interesting to observe in Figure 4 that for healthy patients, or ones with almost no reduction in the aortic valve area (AVA), there is no modification in the flow rate, and thus the pressure drop does not change significantly. Nevertheless, in more severe cases there is an abrupt increase in pressure drop and a decrease in the systolic flow rate. Equation (8) represents this behavior. Moreover, one can infer that there would be an increase in the workload of the heart and, hence, an increase in myocardium size associated with hypertrophy since the heart has to keep its work performance level.

(8) $${\dot{V}}_{syst}=91.28ln\left(AVA\right)+241.02$$

Using these experimental data, we applied the First Law of Thermodynamics in its general form according to Equation (9). This equation can be simplified for the control volume around the human heart as specified in Equation (10). Using this last equation, we estimated an increase in the blood temperature associated with the physiological mechanism of the human heart. According to the articles of Pennes [39] and Chen and Holmes [40], the temperature change in the blood due to its passing through the heart is insignificant. Nevertheless, we will assess it in order to apply the Second Law of Thermodynamics properly.

(9) $$\frac{dE_{CV}}{dt}=\sum_{i}{\dot{m}}_{i}\left(h_{i}+gz_{i}+\frac{V_{i}^{2}}{2}\right)-\sum_{e}{\dot{m}}_{e}\left(h_{e}+gz_{e}+\frac{V_{e}^{2}}{2}\right)+{\dot{Q}}_{CV}-{\dot{W}}_{CV}$$

(10) $$\rho_{bl}{\dot{V}}_{syst}c_{bl}\left(Te-Ti\right)=\rho_{bl}{\dot{V}}_{syst}\left(\frac{V_{i}^{2}}{2}-\frac{V_{e}^{2}}{2}+v_{bl}P_{i}-v_{bl}P_{e}\right)+{\dot{M}}_{Heart}-{\dot{W}}_{Heart}$$

### 2.2. Exergy Analysis

We linked each term in the First Law of Thermodynamics to its analogous counterpart in the exergy analysis in order to apply the exergy analysis to the human body. The general form is shows in Equation (11). For a given reference $P_{0}$, $T_{0}$, and $\phi_{0}$, the exergy of a flow rate is determined by $b=h-h_{0}-T_{0}\left(S-S_{0}\right)$, where the subscript 0 specifies that the property is evaluated at the reference state.

(11) $$\begin{array}{cc} \frac{dB_{CV}}{dt}= & \sum_{i}{\dot{m}}_{i}\left(b_{i}+gz_{i}+\frac{V_{i}^{2}}{2}\right)-\sum_{e}{\dot{m}}_{e}\left(b_{e}+gz_{e}+\frac{V_{e}^{2}}{2}\right)\\ & +\sum_{k}{\dot{Q}}_{k}\left(1-\frac{T_{0}}{T_{k}}\right)-{\dot{W}}_{CV}-{\dot{B}}_{d} \end{array}$$

The term related to the exergy variation of the reactions of oxidation (${\dot{B}}_{M}$) within the human metabolism is calculated according to Equation (12) [29].

(12) $${\dot{B}}_{M_{Heart}}=9501{\dot{m}}_{O_{2}}+3963{\dot{m}}_{CO_{2}}$$

We used Equation (13) to properly evaluate the exergy associated with the heat transfer of the metabolism (${\dot{B}}_{Q_{M,Heart}}$), where the heat transfer associated with the metabolism ${\dot{Q}}_{M_{Heart}}$ was calculated using Equation (5) [29].

(13) $${\dot{B}}_{Q_{M,Heart}}={\dot{Q}}_{M_{Heart}}\left(1-\frac{T_{0}}{T_{Heart}}\right)$$

We quantified the exergy variation of the flow rates entering and leaving the heart using Equation (14), where the term $\Delta {\dot{B}}_{blood}$ stands for the exergy increase of the blood associated with the physical exergy ($\Delta {\dot{B}}_{physical}$) and kinetic exergy ($\Delta {\dot{B}}_{kinetic}$).

(14) $$\Delta {\dot{B}}_{blood}=\Delta {\dot{B}}_{physical}+\Delta {\dot{B}}_{kinetic}$$

The physical exergy is determined using the expression $b=h-h_{0}-T_{0}\left(s-s_{0}\right)$, where blood is considered to be an incompressible fluid subject to an increase in pressure and a temperature gradient. As such, the entropy variation of the blood is assessed through the Gibbs relation: Tds=dh−vdp. Under these conditions, we obtained Equation (15) for the physical exergy [21].

(15) $$\Delta {\dot{B}}_{physical}=\left(\frac{{\dot{V}}_{bl}}{v_{bl}}\right)\left[c_{bl}\left(T_{e}-T_{i}\right)-T_{0}c_{bl}ln\left(\frac{T_{e}}{T_{i}}\right)-\frac{T_{0}v_{bl}}{T_{Heart}}\left(P_{e}-P_{i}\right)\right]$$

The exergy variation of the kinetic exergy of the blood is defined as the kinetic energy variation, as shown in Equation (16) [21].

(16) $$\Delta {\dot{B}}_{kinetic}=\left(\frac{{\dot{V}}_{bl}^{3}}{2v_{bl}}\right)\left(\frac{1}{A_{e}^{2}}-\frac{1}{A_{i}^{2}}\right)$$

Each valve has a head loss where pressure is transformed into internal energy [19]. As such, we calculated the destroyed exergy in each valve as a function of the pressure drop TPG (or ΔP, shown in Figure 3) using Equation (17) [19].

(17) $${\dot{B}}_{d,valve}=\left(\frac{T_{0}}{T_{int}}\right){\dot{V}}_{bl}\Delta P$$

We calculated the destroyed exergy within the human heart using Equation (18). The exergy input is metabolic exergy, of which a portion is destroyed in the four valves of the heart, and the remaining share is transferred as performed power by the heart and an exergy increase of the blood. The heat associated with the metabolism is an order of magnitude lower than the other terms in the analysis [29].

(18) $${\dot{B}}_{d}^{heart}=\sum {\dot{B}}_{d,valve}+{\dot{B}}_{M,Heart}+{\dot{B}}_{Q_{M}}-{\dot{W}}_{Heart}-\Delta {\dot{B}}_{blood}$$

Finally, we assessed exergy efficiency according to Equations (19) and (20). The first equation is traditionally used with the First Law perspective in medical literature [22,23] since the approximation $M\approx B_{M}$ is well established in literature [29]. The idea behind this equation is that the useful output is ${\dot{W}}_{Heart}$. In this article we propose, by means of Equation (20), that a possible definition of the “product” of the heart is the exergy increase of the blood $\Delta {\dot{B}}_{blood}$.

(19) $$\eta_{1}=\frac{{\dot{W}}_{Heart}}{{\dot{B}}_{M,Heart}}$$

(20) $$\eta_{2}=\frac{\Delta {\dot{B}}_{blood}}{{\dot{B}}_{M,Heart}}$$

## 3. Results and Discussions

**Figure 5:**
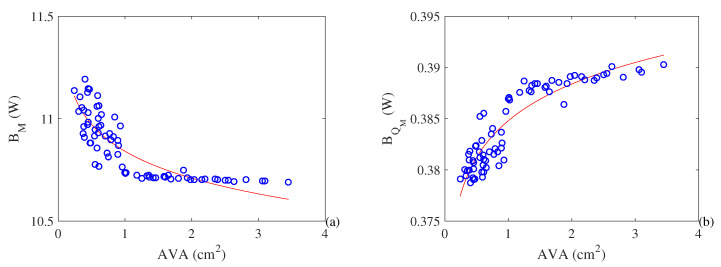
(**a**) Metabolic exergy of the heart (W). (**b**) Exergy associated with the heat released by the metabolism of the heart (W) as a function of the aortic valve area (cm^2^).

We used data from 40 patients with stenosis and 32 points of operation of prostheses, representing a range from healthy patients to clinical suggestions of minor stenosis, to calibrate the thermodynamic and phenomenological model of the human heart. Figure 5 shows the metabolic exergy (${\dot{B}}_{M}$) and the exergy heat released by the body associated with metabolism (${\dot{B}}_{Q_{M}}$) as a function of the severity of the stenosis in the arterial valve. The variable used to represent this phenomenon is the aortic valve area (AVA). Higher values of AVA indicate a healthy subject, whereas lower values indicate an increase in the severity of stenosis. We observe that the metabolism is increasing with respect to the severity of stenosis, reaching values as high as 11 W. However, there is a decrease in the heat associated with metabolism.

**Figure 6:**
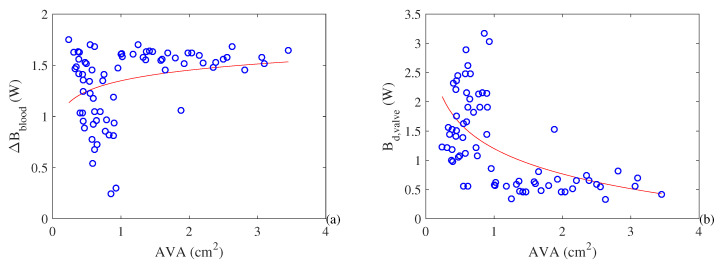
(**a**) Exergy variation of the blood flow rate (W). (**b**) Destroyed exergy in the mitral and arterial valves (W) as a function of the aortic valve area (cm^2^).

As for the increase in the quality of the energy (exergy) of the blood due to performed work, as shown in Figure 6, we note that a reduction in the aortic valve area reduces the exergy increase of the blood (Figure 6a) This is related to an increase in the destroyed exergy rate in the valves (Figure 6b). In both cases there is an asymptotic trend in healthy subjects where a reduction in the valve area does not significantly alter the exergy behavior of the heart.

**Figure 7:**
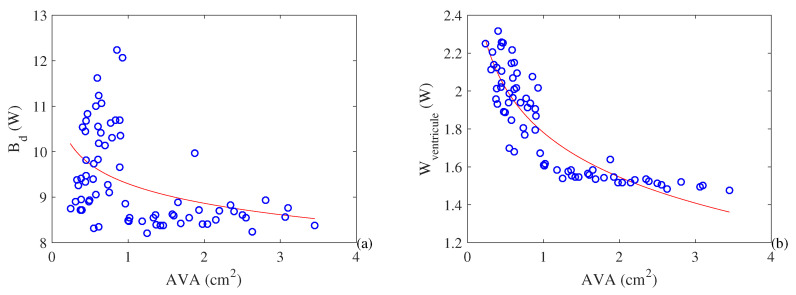
(**a**) Destroyed exergy rate (W) and (**b**) performed power by the heart (W) as a function of the aortic valve area (cm^2^)

Figure 7 shows the heart’s destroyed exergy rate and performed power. It is interesting to highlight that 12 W of exergy destruction is about 10% of the total basal metabolic rate for the whole body [41]. As such, this amount of exergy destruction overcharges the heart, which can be observed in the increased size of the left ventricle in such cases. This ventricular hypertrophy is related to an increase in the cardiac effort as shown in Figure 7b. The increase in the destroyed exergy is around 32% of the values obtained in healthy patients.

**Figure 8:**
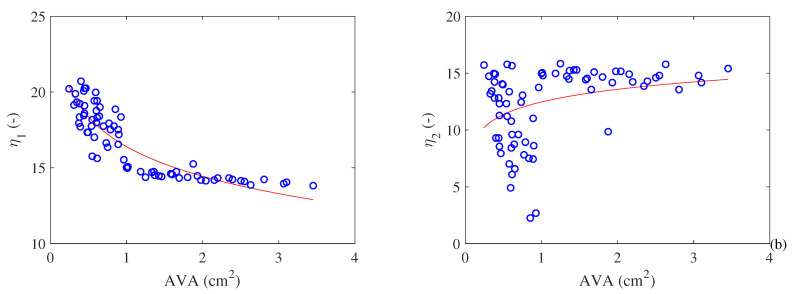
(**a**) Exergy efficiency according to Equation (19) (which is similar to the First Law efficiency). (**b**) Exergy efficiency calculated with Equation (20) as a function of the aortic valve area (cm^2^)

Finally, we assessed the exergy efficiency using two approaches, as indicated in Figure 8. The first one ($\eta_{1}$) is found elsewhere in the medical literature but on an energy basis. It is defined by the ratio of cardiac effort over the metabolism (here on an exergy basis), as shown in Equation (19). Both energy and exergy efficiencies result in similar values since $\dot{M}\approx {\dot{B}}_{M}$. Results showed an increase in the efficiency as a function of work. This is caused by a disproportionate increase in cardiac work due to the reduction in the aortic valve area when compared with the increase in metabolism. Results are in the same order of magnitude of the ones found in the literature [22]. One possible explanation for this behavior is that considering the heart power (${\dot{W}}_{Heart}$) as a useful output is not appropriate (from the combined perspective of the First and Second Laws). When the Second Law of Thermodynamics is applied, one obtains the real increase in the capacity of the blood to perform work, or $\Delta B_{blood}$. Therefore, a conclusion of the present article is that in the first expression of efficiency the performed power does not properly quantify the useful output of pumping. To this aim, Figure 8b shows the results of the exergy efficiency proposed in this article according to Equation (20). In this case, an increase in the restriction of aortic valve area reduces the exergy efficiency indicated as ($\eta_{2}$). Therefore, the increase in the capability of the blood to perform any kind of work decreases as a function of the severity of the pathology. There is a larger increase in the exergy used to increase the quality of the blood and, hence, the performed power of the heart. This physical quantity becomes larger, although the desired outcome (exergy variation of the blood) decreases as the stenosis severity is more prominent.

## 4. Concluding Remarks

In the present article, we proposed a model that took into consideration the many physiological interactions between the cardiovascular system and its surroundings inside the organism. The results are in accordance with previous similar analyses in the literature. A distinguished feature of this article is the use of empirical data from the medical literature for healthy and unhealthy (stenotic) subjects. As a limitation of the present work, there is a lack of experimental results for a healthy valve. This presents itself as an opportunity for future research regarding the simulation of the blood flow in the heart to properly assess the quality of the energy conversion process within the human heart [42].

From this analysis, it is possible to conclude that:

- Both metabolism and cardiac work increase with the increase in the severity of stenosis. T his result explains the hypertrophy that occurs in a pathological heart. Moreover, it is possible to observe an exergy efficiency ($\eta_{2}$) increase with the severity of the pathology. In the other hand, the exergy efficiency ($\eta_{1}$) based of literature (similar to energy efficiency) increases with the severity of the pathology, which was an unexpected result;
- At the same time, the exergy variation of the blood decreases with the restriction of the AVA (i.e., the quality of the energy variation decreases). Therefore, there is a higher cardiac effort leading to higher losses and higher values of destroyed exergy in the valves. This leads to a decrease in exergy efficiency as the pathology evolves;
- This result may be used to help the medical literature to understand the physiological responses of the heart caused by a pathology—in this case a stenosis in the aortic valve area. The main idea is to use the exergy efficiency as an indicator of the degree of severity of the pathology since its behavior is a consequence of several physiological conditions. Therefore, a modification in the hemodynamics of the blood in the valves causes changes in the Second Law efficiency, which differentiates this physical quantity from the First Law efficiency.

## Abbreviations

The following abbreviations are used in this manuscript:

*A*	Area (m^2^)
AVA	aortic valve area (cm^2^)
**B**	Exergy of the control volume (J)
$\dot{B}$	Exergy rate (W)
*b*	Specific exergy (kJ/kg)
*E*	Energy of the control volume (J)
EOA	Effective orifice area (cm^2^)
*g*	Gravitational acceleration (m/s^2^)
$\dot{H}$	Enthalpy rate (W)
*h*	Specific enthalpy (kJ/kg)
$\dot{m}$	Mass flow rate (kg/s)
$\dot{M}$	Energy metabolism (W)
MVA	Mitral valve area (cm^2^)
*P*	Pressure (Pa, mmHg)
$\dot{Q}$	Heat transfer rate (W)
RQ	Respiratory quotient (-)
$\dot{S}$	Entropy rate (W/K)
*s*	Specific entropy (kJ/kgK)
*T*	Temperature (°C)
*t*	Time (s)
TPG	Transvalvular pressure gradient (mm)
$\dot{V}$	Volumetric flow rate (m^3^/s)
*V*	Volume (m^3^)
*v*	Specific volume (m^3^/s)
greek letters:	
η	Efficiency (-)
ρ	Specific mass (kg/m^3^)
subscripts:	
0	Reference state
bl	Blood
CV	Control volume
*d*	Destroyed
*e*	Exit
Heart	Associated with the heart
*i*	Inlet
*M*	Metabolic
$Q_{M}$	Associated with the metabolic heat
syst	systolic
valve	Associated with the valve
